# Progress Toward Regional Measles Elimination — Worldwide, 2000–2018

**DOI:** 10.15585/mmwr.mm6848a1

**Published:** 2019-12-06

**Authors:** Minal K. Patel, Laure Dumolard, Yoann Nedelec, Samir V. Sodha, Claudia Steulet, Marta Gacic-Dobo, Katrina Kretsinger, Jeffrey McFarland, Paul A. Rota, James L. Goodson

**Affiliations:** ^1^Department of Immunization, Vaccines, and Biologicals, World Health Organization, Geneva, Switzerland; ^2^Global Immunization Division, Center for Global Health, CDC; ^3^Division of Viral Diseases, National Center for Immunization and Respiratory Diseases, CDC.

In 2010, the World Health Assembly (WHA) set the following three milestones for measles control to be achieved by 2015: 1) increase routine coverage with the first dose of measles-containing vaccine (MCV1) among children aged 1 year to ≥90% at the national level and to ≥80% in every district, 2) reduce global annual measles incidence to less than five cases per 1 million population, and 3) reduce global measles mortality by 95% from the 2000 estimate* (*1*). In 2012, WHA endorsed the Global Vaccine Action Plan,^†^ with the objective of eliminating measles^§^ in five of the six World Health Organization (WHO) regions by 2020. This report updates a previous report (*2*) and describes progress toward WHA milestones and regional measles elimination during 2000–2018. During 2000–2018, estimated MCV1 coverage increased globally from 72% to 86%; annual reported measles incidence decreased 66%, from 145 to 49 cases per 1 million population; and annual estimated measles deaths decreased 73%, from 535,600 to 142,300. During 2000–2018, measles vaccination averted an estimated 23.2 million deaths. However, the number of measles cases in 2018 increased 167% globally compared with 2016, and estimated global measles mortality has increased since 2017. To continue progress toward the regional measles elimination targets, resource commitments are needed to strengthen routine immunization systems, close historical immunity gaps, and improve surveillance. To achieve measles elimination, all communities and countries need coordinated efforts aiming to reach ≥95% coverage with 2 doses of measles vaccine (*3*).

## Immunization Activities

WHO and the United Nations Children’s Fund (UNICEF) use data from administrative records and vaccination coverage surveys reported annually to estimate MCV1 and second dose (MCV2) coverage through routine immunization services.^¶^ During 2000–2018, estimated MCV1 coverage increased globally from 72% to 86% (Table), although coverage has remained at 84%–86% since 2010, with considerable regional variation. Since 2016, MCV1 coverage has remained relatively constant in the African Region (AFR) (74%–75%), the Eastern Mediterranean Region (EMR) (82%–83%), and the South-East Asia Region (SEAR) (88%–89%); and it has remained constant since 2008 in the European Region (EUR) (93%–95%) and in the Western Pacific Region (WPR) (95%–97%). Estimated MCV1 coverage in the Region of the Americas (AMR) decreased from 92% in 2016 to 88% in 2017 and increased to 90% in 2018.

**TABLE:** Estimates of coverage with the first and second doses of measles-containing vaccine administered through routine immunization services, reported measles cases and incidence, and estimated measles cases and deaths,* by World Health Organization (WHO) region — worldwide, 2000 and 2018

WHO region/ Year (no. of countries in region)	% MCV1^†^ coverage	% countries with ≥90% MCV1 coverage	% MCV2^†^ coverage	% of reporting countries with <5 measles cases per 1 million	No. of reported measles cases^§^	Measles incidence per 1 million^§,¶^	Estimated no. of measles cases (95% CI)	Estimated no. of measles deaths (95% CI)	Estimated % measles mortality reduction, 2000–2018	Cumulative no. of measles deaths averted by vaccination, 2000–2018
**African**										
2000 (46)	53	9	5	8	520,102	836	10,723,800 (7,718,000–17,119,100)	345,600 (236,300–562,100)	85	12,146,900
2018 (47)	74	30	26	47	125,426	118	1,759,000 (1,141,200–6,002,100)	52,600 (32,000–173,400)		
**Americas**										
2000 (35)	93	63	65	89	1,754	2	8,770 (4,400–35,100)	NA**	NA	97,100
2018 (35)	90	57	82	91	16,327	24	83,500 (41,800–334,200)	NA		
**Eastern Mediterranean**										
2000 (21)	71	57	28	17	38,592	90	2,427,900 (1,503,800–3,892,900)	37,900 (21,700–64,000)	−29	2,820,600
2018 (21)	82	57	74	35	64,722	93	2,852,700 (2,293,700–4,265,200)	49,000 (36,700–72,500)		
**European**										
2000 (52)	91	62	48	45	37,421	50	860,176 (227,200–6,668,300)	400 (100–2,200)	50	95,600
2018 (53)	95	89	91	34	82,523	98	861,800 (71,100–6,480,300)	200 (0–1,800)		
**South-East Asia**										
2000 (10)	63	30	3	0	78,558	51	11,411,900 (8,764,600–15,572,100)	141,700 (100,100–199,600)	72	6,825,400
2018 (11)	89	82	80	36	34,741	18	3,803,800 (2,856,700–6,702,900)	39,100 (24,800–76,000)		
**Western Pacific**										
2000 (27)	85	48	2	30	177,052	105	2,786,500 (1,923,900–22,167,600)	10,000 (5,200–74,200)	87	1,213,200
2018 (27)	95	59	91	77	29,497	15	408,400 (42,500–16,753,800)	1,300 (100–2,786,500)		
**Total**										
**2000 (191)**	**72**	**45**	**18**	**38**	**853,479**	**145**	**28,219,100 (20,141,900–65,455,000)**	**535,600 (363,400–901,700)**	**73**	**23,198,800**
**2018 (194)**	**86**	**61**	**69**	**54**	**353,236**	**49**	**9,769,400 (6,446,900–40,538,500)**	**142,300 (93,600–387,900)**		

**Abbreviations:** CI = confidence interval; MCV1 = first dose of measles-containing vaccine; MCV2 = second dose of measles-containing vaccine; NA = not applicable; UNICEF = United Nations Children's Fund.

* Mortality estimates for 2000 might be different from previous reports. When the model used to generate estimated measles deaths is rerun each year using new WHO/UINICEF estimates of national immunization coverage (WUENIC) data, as well as updated surveillance data, adjusted results for each year, including the baseline year, are also produced and updated.

^†^ Coverage data: WUENIC. Geneva, Switzerland: World Health Organization; 2019. https://www.who.int/immunization/monitoring_surveillance/data/en.

^§^ Reported measles cases (2018) from World Health Organization. Geneva, Switzerland: World Health Organization; 2019. https://apps.who.int/immunization_monitoring/globalsummary/timeseries/tsincidencemeasles.html.

^¶^ Cases per 1 million population; population data from United Nations, Department of Economic and Social Affairs, Population Division, 2019. Any country not reporting data on measles cases for that year was removed from both the numerator and denominator.

** Estimated measles mortality was too low to allow reliable measurement of mortality reduction.

Globally, 118 (61%) countries achieved ≥90% MCV1 coverage in 2018, an increase from 86 (45%) countries in 2000, but a decrease from 126 (65%) countries during 2012–2013. In 2018, MCV1 coverage was ≥95% nationally in 78 (40%) countries and ≥80% in all districts in 57 (29%) countries.** In 2018, 19.2 million infants worldwide did not receive MCV1 through routine immunization services. The six countries with the most unvaccinated infants were Nigeria (2.4 million), India (2.3 million), Pakistan (1.4 million), Ethiopia (1.3 million), Indonesia (1.2 million), and the Philippines (0.7 million).

Estimated MCV2 coverage increased globally from 18% in 2000 to 69% in 2018, largely because of an increase in the number of countries providing MCV2 from 98 (51%) in 2000 to 171 (88%) in 2018 (Table). Four countries (Bolivia, the Dominican Republic, Honduras, and the Solomon Islands) introduced MCV2 in 2018.

In 2018, approximately 346 million persons received measles vaccination during 45 supplementary immunization activities (SIAs)^††^ in 37 countries; India’s 2018 SIA accounted for 47% of all persons vaccinated in SIAs worldwide. An additional 13 million persons were vaccinated during measles outbreak response activities.

## Reported Measles Incidence

In 2018, all 194 WHO member countries conducted measles surveillance, and 191 (98%) had access to standardized quality-controlled laboratory testing through the WHO Global Measles and Rubella Laboratory Network. However, surveillance remains weak in many countries, and only 84 (55%) of 152 countries that reported surveillance indicators achieved the sensitivity indicator target of ≥2 discarded measles and rubella^§§^ cases per 100,000 population.

**FIGURE 1:**
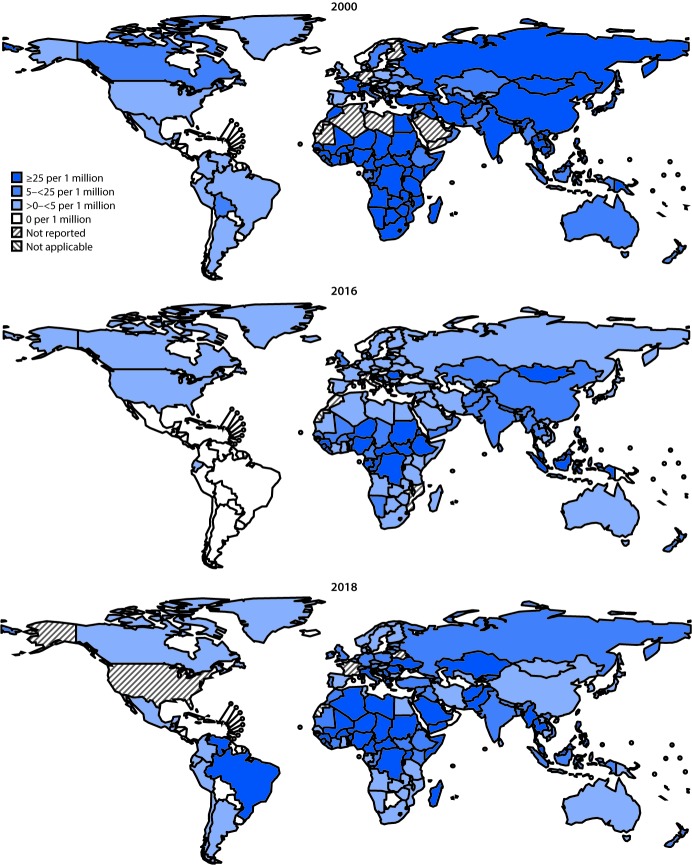
Reported measles incidence per 1 million persons — worldwide, 2000, 2016, and 2018

Countries report the number of incident measles cases^¶¶^ to WHO and UNICEF annually using the Joint Reporting Form.*** During 2000–2018, the number of reported cases decreased 59%, from 853,479 in 2000 to 353,236 in 2018, and measles incidence decreased 66%, from 145 to 49 cases per million population (Table). However, compared with the reported number of cases (132,413) and incidence (19 cases per million) in 2016, both cases and incidence increased in 2018, the highest levels since 2011 (Figure 1). Compared with 2016, the number of measles cases increased 167% globally, including increases of 246% in AFR, 16,732% in AMR, 931% in EMR, 1,791% in EUR, and 26% in SEAR.^†††^ In WPR, the number of measles cases decreased 49%, primarily because of decreased cases in China. In 2018, five (3%) of 179 reporting countries (Democratic Republic of the Congo, Liberia, Madagascar, Somalia, and Ukraine) had measles incidences >600 per million and accounted for 45% (157,239 cases) of all reported cases worldwide. The percentage of reporting countries with annual measles incidence of <5 cases per million population increased from 38% (64 of 169) in 2000 to 70% (125 of 178) in 2016, then decreased to 54% (96 of 179) in 2018 (Table) (Figure 1).

Genotypes of viruses isolated from measles cases were reported by 95 (73%) of 131 countries reporting at least one measles case in 2018. Among the 24 recognized measles virus genotypes, 11 were detected during 2005–2008, eight during 2009–2014, six in 2016, five in 2017, and four in 2018 (*4*). In 2018, among 7,155 reported virus sequences, 3,011 (42%) were genotype B3; 20 (0.3%) were D4; 3,774 (53%) were D8; and 350 (5%) were H1.

## Measles Case and Mortality Estimates

**FIGURE 2:**
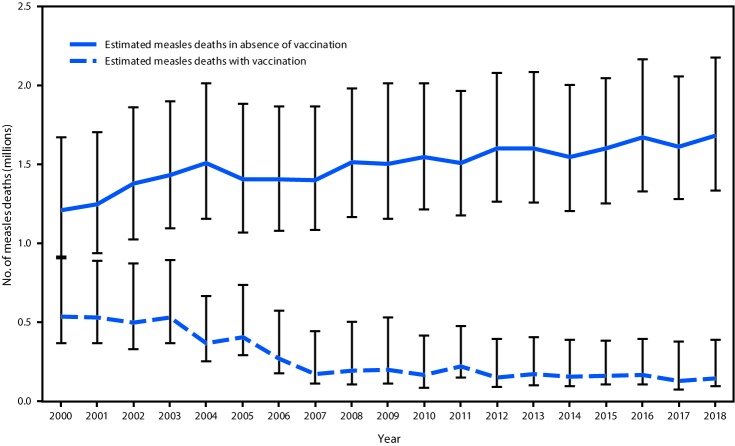
Estimated annual number of measles deaths, by vaccination status — worldwide, 2000–2018* * Deaths prevented by vaccination are estimated by the area between estimated deaths with vaccination and those without vaccination (cumulative total of 23.2 million deaths prevented during 2000–2018). Error bars represent upper and lower 95% confidence limits around the point estimate.

A previously described model for estimating measles cases and deaths was updated with new measles vaccination coverage data, case data, and United Nations population estimates for all countries during 2000–2018, enabling derivation of a new series of disease and mortality estimates (*5*). For countries with anomalous estimates in previous iterations, the model was modified slightly to generate mortality estimates consistent with observed case data. Based on the updated data, the estimated number of measles cases decreased 65%, from 28,219,100 (95% confidence interval [CI] = 20,141,900–65,455,000) in 2000 to 9,769,400 (95% CI = 6,446,900–40,538,500) in 2018. During this period, estimated measles deaths decreased 73%, from 535,600 (95% CI = 363,400–901,700) to 142,300 (95% CI = 93,600–387,900) (Table) (Figure 2). During 2000–2018, compared with no measles vaccination, measles vaccination prevented an estimated 23.2 million deaths globally.

## Regional Verification of Measles Elimination

By the end of 2018, 82 (42%) countries had been verified as having eliminated measles. Austria, Bahrain, North Korea, Oman, Singapore, Switzerland, and Timor-Leste were verified as having achieved elimination during 2018. No AFR country had yet been verified as having eliminated measles. In the AMR, a region that had achieved verification of measles elimination in 2016, endemic measles transmission was reestablished in Venezuela in 2018 and in Brazil in 2019. In EUR, endemic measles transmission was reestablished during 2018 in Albania, Czechia, Greece, and the United Kingdom.

## Discussion

During 2000–2018, increased coverage with MCV1 and MCV2, widespread SIAs, and other elimination efforts contributed to a 66% decrease in reported measles incidence, a 73% reduction in estimated measles mortality, and a reduction in the number of circulating measles virus genotypes worldwide. Despite this progress, the 2015 global milestones were not met: MCV1 coverage has stagnated for nearly a decade, MCV2 coverage is only 69%, and suboptimal surveillance limits data-driven actions. Reported measles incidence has increased in five regions since 2016 and estimated global measles mortality has increased since 2017. Increased measles cases and outbreaks occurred mostly among unvaccinated persons, including school-aged children and young adults.

The causes of the measles resurgence during 2017–2018 are multifactorial and vary by country. Large sustained outbreaks in a few countries with weak immunization systems accounted for most reported measles cases during this time. In addition, unidentified or unaddressed immunity gaps in older children and adults, because of historically weak routine immunization programs and inadequate SIA coverage, led to sustained transmission in some countries that previously had low incidence or had eliminated measles (*6*). As well, international travel by infected persons, including both unimmunized foreign visitors and unimmunized residents traveling abroad and returning home, facilitated international spread of measles. For example, in 2018, Israel experienced nearly 100 measles importations from multiple countries including Philippines, Ukraine, and the United Kingdom; and importations from Israel and Ukraine led to outbreaks in the United States (*7*). Sustaining elimination in the face of frequent importations and gaps in vaccination coverage presents challenges. For example, after having experienced >100 importations in 2018 as a consequence of inadequate vaccination coverage, endemic measles virus transmission has been reestablished in the United Kingdom. Countries such as Cambodia, which, through sustained efforts, identified and closed immunity gaps to achieve elimination, but which border countries with ongoing endemic transmission, must remain vigilant to identify and stop measles outbreaks rapidly. Before international travel, travelers from all countries should ensure they have been appropriately vaccinated against measles. Progress toward measles elimination will regress without a unified effort by all communities and countries.

Evaluations of routine immunization programs to identify barriers to vaccination indicate that children miss MCV1 and MCV2 doses for many reasons, including families’ limited awareness of the need for vaccination, limited access to or financial barriers to receiving vaccination; vaccine stock-outs; political instability; and vaccine hesitancy and misinformation. WHO’s Global Routine Immunization Strategies and Practices and The Guide to Tailoring Immunization Programmes provides guidance on identifying demand and supply barriers to routine vaccination and strengthening immunization programs (*8*,*9*). Outbreaks should serve as opportunities to investigate underlying causes of undervaccination and to design specific routine immunization strengthening activities to prevent future outbreaks. In addition, population immunity gaps should be identified through triangulation of data, including surveillance and vaccination coverage data, and should be targeted by vaccination activities.

The findings in this report are subject to at least two limitations. First, large differences between estimated and reported incidence indicate overall low surveillance sensitivity, making comparisons between regions difficult to interpret. Second, the measles mortality model estimates might be affected by biases in model inputs, including vaccination coverage and surveillance data.

The trends of increasing measles incidence and mortality are reversible; however, further progress toward achieving elimination goals will require 1) resource commitments to strengthen routine immunization systems, close historical immunity gaps, and improve surveillance to rapidly detect and respond to cases, and 2) a new perspective to use measles as a stimulus and guide to improving immunization programs. To achieve measles elimination, all communities and countries need coordinated efforts aiming to reach ≥95% coverage with 2 doses of measles vaccine.

As the period covered by the Global Vaccine Action Plan 2012–2020 approaches its end, a new vision and strategy for accelerated progress on immunization for 2021–2030 is being developed by countries and stakeholders (*10*). Pillars of this evolving strategy include commitment and demand, research and innovation, life course and integration, and supply and sustainability; all of these are vital to achieving and maintaining measles elimination. This new agenda should be used to secure the necessary resource commitments to improve coverage and equity substantially and, in so doing, further progress toward achieving the measles elimination goals.

**Summary**

**What is already known about this topic?**

In 2012, the World Health Assembly endorsed the Global Vaccine Action Plan; countries in all six World Health Organization regions have adopted goals to eliminate measles by 2020.

**What is added by this report?**

During 2000–2018, annual reported measles incidence decreased 66%, and annual estimated measles deaths decreased 73%. Since 2000, measles vaccination has prevented an estimated 23.2 million deaths globally. However, measles incidence increased in five regions during 2016–2018.

**What are the implications for public health practice?**

To achieve regional measles elimination goals, resource commitments are needed to strengthen routine immunization systems, close immunity gaps, and improve case-based surveillance.

## Footnotes

* The coverage milestone is to be met by every country, whereas the incidence and mortality reduction milestones are to be met globally.

† The Global Vaccine Action Plan is the implementation plan of the Decade of Vaccines, a collaboration between WHO; UNICEF; the Bill and Melinda Gates Foundation; the National Institute of Allergy and Infectious Diseases; the African Leaders Malaria Alliance; Gavi, the Vaccine Alliance; and others to extend the full benefit of immunization to all persons by 2020 and beyond. In addition to 2015 targets, it also set a target for measles and rubella elimination in five of the six WHO regions by 2020. https://www.who.int/immunization/global_vaccine_action_plan/en; https://apps.who.int/gb/ebwha/pdf_files/wha65/a65_22-en.pdf.

§ Measles elimination is defined as the absence of endemic measles virus transmission in a region or other defined geographic area for ≥12 months, in the presence of a high-quality surveillance system that meets targets of key performance indicators.

¶ For MCV1, among children aged 1 year or, if MCV1 is given at age ≥1 year, among children aged 24 months. For MCV2, among children at the recommended age for administration of MCV2, per the national immunization schedule. WHO/UNICEF estimates of national immunization coverage are available at https://www.who.int/immunization/monitoring_surveillance/data/en.

** In 2000, 191 countries were requested to report to WHO; by 2018, 194 member states were requested to report because of the creation of new countries. For district level coverage, only countries that reported data are in the numerator, whereas the denominator is all WHO countries in that year (191–194) regardless of whether they reported data.

†† Supplementary immunization activities (SIAs) generally are carried out using two target age ranges. An initial, nationwide catch-up SIA focuses on all children aged 9 months–14 years, with the goal of eliminating susceptibility to measles in the general population. Periodic follow-up SIAs then focus on all children born since the last SIA. Follow-up SIAs generally are conducted nationwide every 2–4 years and focus on children aged 9–59 months; their goal is to eliminate any measles susceptibility that has developed in recent birth cohorts due to low MCV coverage and to protect children who did not respond to MCV1. Data on SIAs by country are available at https://www.who.int/immunization/monitoring_surveillance/data/Summary_Measles_SIAs.xls?ua=1.

§§ A discarded case is defined as a suspected case that has been investigated and determined not to be measles or rubella using 1) laboratory testing in a proficient laboratory or 2) epidemiological linkage to a laboratory-confirmed outbreak of a communicable disease that is not measles or rubella. The discarded case rate is used to measure the sensitivity of measles surveillance.

¶¶ https://apps.who.int/immunization_monitoring/globalsummary/timeseries/tsincidencemeasles.html; data reported here as of July 15, 2019. Only countries that reported data are in the numerator, whereas the denominator is all WHO countries in that year (191–194) regardless of whether they reported data.

***
https://www.who.int/immunization/monitoring_surveillance/routine/reporting/en/ .

††† Twenty-five countries did not report case data in 2000: Algeria, Austria, Belgium, Comoros, Equatorial Guinea, Fiji, Finland, Germany, Guinea-Bissau, Ireland, Libya, Mauritania, Monaco, Montenegro, North Korea, Samoa, Saudi Arabia, Seychelles, Slovenia. Solomon Islands, South Sudan. Switzerland, Timor-Leste, Tuvalu, and Yemen. Sixteen countries did not report case data in 2016: Belgium, Cabo Verde, Cook Islands, Haiti, Ireland, Italy, Kiribati, Marshall Islands, Monaco, Morocco, Mozambique, Niue, Samoa, Singapore, Tuvalu, and Vanuatu. Fifteen countries did not report case data in 2018: Belarus, France, Israel, Kuwait, Luxembourg, Marshall Islands, Mauritius, Montenegro, Nauru, Niue, North Macedonia, Palau, Seychelles, Tuvalu, and United States. Countries do not provide WHO with their reasons for not reporting case data.
